# Metabolism and Regulatory Functions of *O*-Acetylserine, *S*-Adenosylmethionine, Homocysteine, and Serine in Plant Development and Environmental Responses

**DOI:** 10.3389/fpls.2021.643403

**Published:** 2021-05-07

**Authors:** Mutsumi Watanabe, Yukako Chiba, Masami Yokota Hirai

**Affiliations:** ^1^Graduate School of Biological Science, Nara Institute of Science and Technology, Ikoma, Japan; ^2^Graduate School of Life Sciences, Faculty of Science, Hokkaido University, Sapporo, Japan; ^3^RIKEN Center for Sustainable Resource Science, Yokohama, Japan; ^4^Graduate School of Bioagricultural Sciences, Nagoya University, Nagoya, Japan

**Keywords:** aspartate-family amino acid, homocysteine, *O*-acetylserine, one-carbon metabolism, serine, *S*-adenosylmethionine, sulfur assimilation

## Abstract

The metabolism of an organism is closely related to both its internal and external environments. Metabolites can act as signal molecules that regulate the functions of genes and proteins, reflecting the status of these environments. This review discusses the metabolism and regulatory functions of *O*-acetylserine (OAS), *S*-adenosylmethionine (AdoMet), homocysteine (Hcy), and serine (Ser), which are key metabolites related to sulfur (S)-containing amino acids in plant metabolic networks, in comparison to microbial and animal metabolism. Plants are photosynthetic auxotrophs that have evolved a specific metabolic network different from those in other living organisms. Although amino acids are the building blocks of proteins and common metabolites in all living organisms, their metabolism and regulation in plants have specific features that differ from those in animals and bacteria. In plants, cysteine (Cys), an S-containing amino acid, is synthesized from sulfide and OAS derived from Ser. Methionine (Met), another S-containing amino acid, is also closely related to Ser metabolism because of its thiomethyl moiety. Its S atom is derived from Cys and its methyl group from folates, which are involved in one-carbon metabolism with Ser. One-carbon metabolism is also involved in the biosynthesis of AdoMet, which serves as a methyl donor in the methylation reactions of various biomolecules. Ser is synthesized in three pathways: the phosphorylated pathway found in all organisms and the glycolate and the glycerate pathways, which are specific to plants. Ser metabolism is not only important in Ser supply but also involved in many other functions. Among the metabolites in this network, OAS is known to function as a signal molecule to regulate the expression of OAS gene clusters in response to environmental factors. AdoMet regulates amino acid metabolism at enzymatic and translational levels and regulates gene expression as methyl donor in the DNA and histone methylation or after conversion into bioactive molecules such as polyamine and ethylene. Hcy is involved in Met–AdoMet metabolism and can regulate Ser biosynthesis at an enzymatic level. Ser metabolism is involved in development and stress responses. This review aims to summarize the metabolism and regulatory functions of OAS, AdoMet, Hcy, and Ser and compare the available knowledge for plants with that for animals and bacteria and propose a future perspective on plant research.

## Introduction

Plants (land plants) are photosynthetic auxotrophs that assimilate carbon dioxide and inorganic ions absorbed from the soil into organic compounds. They share common biosynthetic pathways for proteinogenic amino acids with prokaryotic microbes such as *Escherichia coli*. However, plant metabolism is compartmentalized into several organelles that have specific functions. It also differs from the metabolism of animals, as they consume nutrients through their diet.

Land plants have evolved complex body plans, which require developmental regulation of metabolism. They have also evolved adaptive mechanisms that regulate metabolism to cope with terrestrial environments that are harsher than aquatic environments. To understand why plants have evolved extant metabolic systems, a comparison of these systems with those in other organisms will be helpful. In this review, we aim to summarize commonalities and differences in metabolism related to sulfur (S)-containing amino acids and one-carbon (C_1_) metabolism among plants, bacteria, and animals. The changes in metabolite content are due to metabolic regulation, and the metabolites in turn regulate metabolism. We also elucidate the regulatory functions of metabolites closely related to each other, focusing on four amino acids: *O*-acetylserine (OAS), *S*-adenosylmethionine (AdoMet), homocysteine (Hcy), and serine (Ser) (Table 1).

**TABLE 1:** Abbreviations and synonyms used in this article.

	Abbreviations	Synonyms
**Compounds**		
5,10-Methenyl-THF	5,10=CH-THF	
5,10-Methylene-THF	5,10-CH_2_-THF	
5-Methyl-THF	5-CH_3_-THF	
10-Formyl-THF	10-HCO-THF	
1-Aminocyclopropane 1-carboxylate	ACC	
*S*-adenosylhomocysteine	AdoHcy	SAH
*S*-adenosylmethionine	AdoMet	SAM
Adenosine-5′-phosphate	APS	
Cystathionine	CysT	
Decarboxylated AdoMet	dAdoMet	dcSAM
1,2-Dihydro-3-keto-5-methylthiopentene	DHKMP	
Homocysteine	Hcy	Hcys
5′-Methylthioadenosine	MTA	
2-Keto-4-methylthiobutyrate	KMTB	KMBA
5-Methylthioribose	MTR	
5-Methylthioribose-1-phosphate	MTR-P	MTR-1-P
5-Methylthioribulose-1-phosphate	MTRu-P	MTRu-1-P
Nicotianamine	NA	
*N*-acetylserine	NAS	
*O*-acetylserine	OAS	
*O*-phosphohomoserine	OPH	
3′-Phospho-APS	PAPS	
3-Phosphoglycerate	PGA	
*S*-Methylmethionine	SMM	
Tetrahydrofolate	THF	
**Enzymes**		
ACC oxidase	ACO	
Arg decarboxylase	ADC	
Adenosine kinase	ADK	
AdoMet decarboxylase	AdoMetDC	SAMDC
Alanine:hydroxypyruvate (Ser:pyruvate) aminotransferase	AH-AT	
Aspartate kinase	AK	
APS kinase	APK	
APS reductase	APR	
Acidoreductone oxygenase	ARD	
ATP sulfurylase	ATPS	
Cystathionine β-lyase	CBL	
Cdystathionine γ-synthase	CGS	
Cysteine synthase complex	CSC	
Dehydratase-enolase-phosphatase-complex 1	DEP1	
Glycine decarboxylase complex	GDC	
10-Formyl-THF synthetase	FTHFS	
Glycerate dehydrogenase	GDH	
Glycerate kinase	GLYK	
Homocysteine *S*-methyltransferase	Hcy*S*MT	
Histone methyltransferase	HMT	
Hydroxypyruvate reductase	HPR	
Homoserine dehydrogenase	HSDH	
*S*-adenosylmethionine synthetase/methionine adenosyltransferase	MAT	SAM synthase, SAMS
Methionyl-tRNA synthetase	MetRS	
Methionine *S*-methyltransferase	Met*S*MT	
Methionine synthase	MS	
5,10-Methenyl-THF cyclohydrolase	MTHFC	
5,10-Methylene-THF dehydrogenase	MTHFD	
Bifunctional MTHFD/MTHFC	MTHFD/C	
5,10-Methylene-THF reductase	MTHFR	
5-Methylthioribose-1-phosphate isomerase	MTI	
5-Methylthioribose kinase	MTK	
5-Methylthioadenosine nucleosidase	MTN	
*O*-acetylserine-(thiol)-lyase	OAS-TL	OASS
3-Phosphoglycerate phosphatase	PGAP	
3-Phosphoglycerate dehydrogenase	PGDH	
3-Phosphoserine aminotransferase	PSAT	
3-Phosphoserine phosphatase	PSP	
*S*-Adenosylhomocysteine hydrolase	SAHH	
Serine acetyltransferase	SERAT	SAT
Serine hydroxymethyltransferase	SHM	SHMT
Sulfite reductase	SiR	
Spermidine synthase	SPDS	
Spermine synthase	SPMS	
Threonine synthase	TS	
Thermospermine synthase	TSPMS	

**FIGURE 1:**
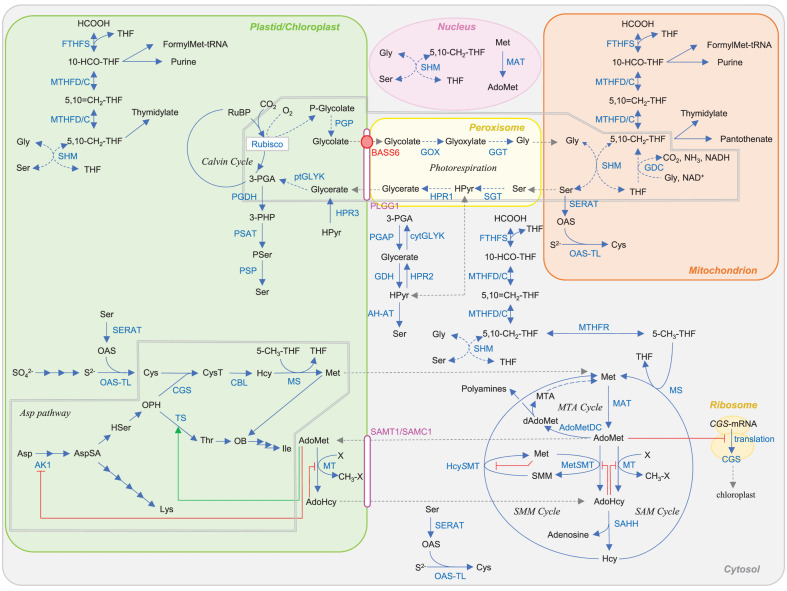
Plant metabolic pathways targeted in this article. Red and green lines indicate repression and activation of the reaction by metabolites. Abbreviations: BASS6, bile acid sodium symporter 6; PLGG1, plastidal glycolate glycerate translocator 1; SAMT1/SAMC1, *S*-adenosylmethionine transporter 1/*S*-adenosylmethionine carrier 1. Other abbreviations are listed in Table 1.

In the first part of this review, we introduce the metabolism of OAS (section “OAS Is a Key Metabolite in Cys Synthesis”), Hcy (section “Hcy Is Essential for *de novo* Met Synthesis and AdoMet Recycling”), and AdoMet (section “AdoMet Provides Methyl and Aminopropyl Moieties and Is Recycled in the Three Cycles”) in the context of S assimilation and S-containing amino acid biosynthesis. We also introduce C_1_ metabolism (section “One-Carbon Metabolism Involves Tetrahydrofolate and AdoMet Metabolism”) and Ser metabolism (section “Ser Metabolism Interconnects Metabolic Network”), which are closely related to the metabolism of OAS, Hcy, and AdoMet. The metabolic pathways described in these chapters are overviewed in Figure 1. In the second part, we describe the regulatory control functions of OAS (section “OAS”), AdoMet (section “AdoMet”), Hcy (section “Hcy”), and Ser (section “Ser”) in plants in comparison to those of bacteria and animals.

## Metabolism Involving OAS, Adomet, Hcy, and Ser

### OAS Is a Key Metabolite in Cys Synthesis

**FIGURE 2:**
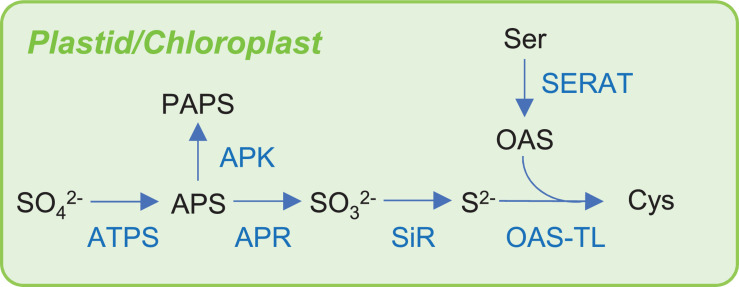
Sulfur assimilation pathway in plant. Abbreviations are summarized in Table 1.

S assimilation of inorganic S into organic S in plants, bacteria, and yeast is especially important in nature because animals do not have these assimilatory mechanisms. Animals require methionine (Met) as an essential amino acid because it is a source of nutrient S (Brosnan and Brosnan, 2006). Sulfate is the principal source of S for plants and many bacteria (Leustek et al., 2000; Takahashi et al., 2011). To incorporate the S atom of sulfate into cysteine (Cys), which is the first organic compound with reduced S, the reduction of sulfate to sulfide is required. Plants universally use the adenosine-5′-phosphate (APS) reductase pathway (Figure 2), whereas bacteria use either the APS reductase (APR) pathway or the 3′-phospho-APS (PAPS) reductase pathway. In plants and sulfate-assimilating bacteria, inorganic sulfate is reduced to APS by ATP sulfurylase (ATPS) and then converted to sulfite by APR, which is reduced to sulfide by sulfite reductase (SiR; Bick et al., 2000). In other bacteria including *E. coli* or in yeast, sulfate is incorporated into APS by ATPS and then converted to PAPS by APS kinase (APK), which is reduced to sulfite by PAPS reductase and subsequently to sulfide by SiR (Masselot and De Robichon-Szulmajste, 1975; Sekowska et al., 2000). The biosynthesis of Cys represents the final step of sulfate assimilation. In bacteria and plants, Cys is synthesized from OAS, which is an activated Ser, by incorporating sulfide into the direct sulfhydrylation pathway (Figure 2 for plants). Ser acetyltransferase (SERAT also called SAT) catalyzes the OAS synthesis from acetyl-CoA and Ser. Synthesized OAS is condensed with sulfide by OAS(thiol)lyase/OAS sulfhydrylase (OAS-TL also called OASS) to form Cys. In yeast, sulfide can be condensed with OAS to produce Cys or with *O*-acetylhomoserine to produce Hcy, which can be converted to cystathionine (CysT) and then to Cys. The budding yeast *Saccharomyces cerevisiae* has only *O*-acetylhomoserine pathway, while the fission yeast *Schizosaccharomyces pombe* has both pathways (Cherest and Surdin-Kerjan, 1992; Marzluf, 1997). Bacteria can also integrate thiosulfate into OAS to produce *S*-sulfocysteine catalyzed by OAS-TL and then Cys (Hulanicka et al., 1979; Nakamura et al., 1984). In bacteria, there are more than 20 genes required for the transport and assimilatory reduction of sulfate and Cys biosynthesis from several operons belonging to a cys regulon regulated by a transcriptional regulator CysB (see section “OAS and *N*-Acetylserine Regulate Transcription in Bacteria and Plants”). Generally, the cytosol is the major site for the assimilation and synthesis of Cys in bacteria. In plants, the sulfate reduction pathway, which produces sulfide, is mainly localized to plastids, whereas the syntheses of OAS and Cys are localized to multiple compartments, including the cytosol, plastids, and mitochondria (Saito, 2004; Takahashi et al., 2011) (Figure 1). The activity distributions of the dominant SERAT and OAS-TL differed between the subcellular compartments, tissues, and species. For *Arabidopsis thaliana* (hereafter *Arabidopsis*), there are five SERAT isoforms and three OAS-TL isoforms, which are distributed over the three compartments (Noji et al., 1998; Wirtz et al., 2001; Kawashima et al., 2005; Haas et al., 2008; Heeg et al., 2008; Watanabe et al., 2008a, b). In *Arabidopsis* leaf, mitochondrial SERAT and cytosolic OAS-TL predominantly contribute to the production of OAS and Cys, respectively, but the dominant contributions seem to differ in other tissues, such as the roots, flower, silique, and seeds, and in different growth conditions (Heeg et al., 2008; Watanabe et al., 2008a, b; Birke et al., 2013). In other species, dominant SERAT activity was detected in the cytosol of *Citrullus vulgaris* seedlings (Saito et al., 1995), in plastids of *Spinacea oleracea* leaf (Brunold and Suter, 1982), and in the mitochondria of *Phaseolus vulgaris* seedlings (Smith, 1972) and *Pisum sativum* leaves (Ruffet et al., 1995; Droux, 2003).

### Hcy Is Essential for *de novo* Met Synthesis and AdoMet Recycling

Cys is further converted to another S-containing proteinogenic amino acid Met in plants and bacteria (Figure 1 for plants). This metabolic pathway comprised three reactions catalyzed by CysT γ-synthase (CGS), CysT β-lyase (CBL), and Met synthase (MS). CGS catalyzes the formation of CysT from Cys and *O*-phosphohomoserine (OPH), whereas CBL generates Hcy from CysT. In the last step of Met synthesis, the methyl group is transferred to an S atom of Hcy by MS using 5-methyltetrahydrofolate (5-CH_3_-THF) as a methyl donor. OPH provides a carbon skeleton of Met and works as the common biosynthetic precursor for threonine (Thr) and isoleucine (Ile). In plants, CGS and CBL are localized in the chloroplast, whereas MS is considered to exist in both the chloroplast and cytosol. Ravanel et al. (2004) reported that a chloroplastic MS isoform of *Arabidopsis* (AtMS3) was responsible for the methylation of Hcy that is synthesized *de novo* in this organelle, whereas two cytosolic MS isoforms (AtMS1 and AtMS2) are involved in the regeneration of Met from Hcy, which is derived from *S*-adenosylhomosysteine (AdoHcy), a product of methylation reactions that use AdoMet. The 5-CH_3_-THF is synthesized by 5,10-CH_2_-THF reductase (MTHFR) from methylene-THF (5,10-CH_2_-THF) and serves as a methyl donor in the reactions catalyzed by MS. However, as the plastids lack MTHFR and cannot generate 5-CH_3_-THF, it is not clear how they might make Met from Hcy (Christensen and MacKenzie, 2006). As AdoHcy inhibits methyltransferase activity (Tehlivets et al., 2013), degradation of AdoHcy by *S*-adenosylhomocysteine hydrolase (SAHH) into Hcy is important to maintain the activity of the methyltransferases involved in various important reactions. SAHH is localized only to the cytosol.

While bacteria have the same biosynthetic pathways for Met, animals cannot produce this amino acid and must acquire it from their diets. In animals, Cys is synthesized from Met via a pathway in which Met is sequentially converted to AdoMet, AdoHcy, Hcy, CysT, and finally Cys (Poloni et al., 2015).

In plant, Met is a member of the aspartate (Asp) family of amino acids because its carbon skeleton is derived from Asp. Among the nine essential amino acids for humans, lysine (Lys), Thr, Ile, and Met are synthesized from Asp in plants and bacteria. The biosynthetic pathway of these Asp-family amino acids (Asp pathway, surrounded by gray double line in Figure 1) in plants is finely regulated via feedback inhibition and stimulation of enzymatic activities by Lys, Thr, Ile, and AdoMet (Jander and Joshi, 2009; Galili et al., 2016). The pathway is also regulated at a translational level by AdoMet (see section “AdoMet Is a Critical Biomolecule Controlling Enzymatic Activity and Gene Expression at the Level of Transcription and Translation”).

### AdoMet Provides Methyl and Aminopropyl Moieties and Is Recycled in the Three Cycles

**FIGURE 3:**
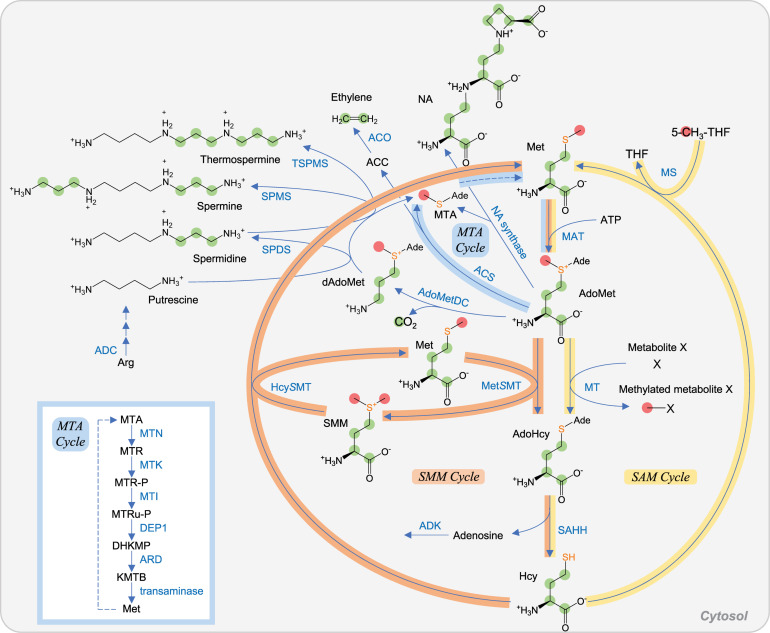
AdoMet metabolism in plant. Carbon atoms of C_1_ unit and those derived from Asp are shown as red and green circles, respectively. Sulfur atoms derived from Cys are highlighted in orange. SAM cycle, SMM cycle, and MTA cycle (also known as the Yang cycle or Met salvage cycle) are indicated in yellow, orange, and pale blue, respectively. Among the reactions of the MTA cycle, six steps from MTA to Met are shown in the lower left box. Abbreviations: Ade, adenosyl group. Other abbreviations are listed in Table 1.

AdoMet is an important molecule that serves as a methyl donor in numerous methylation reactions in all living organisms. The methylation of DNA and histones regulates gene expression and thus various biological phenomena. Decarboxylated AdoMet (dAdoMet) serves as an aminopropyl donor for the synthesis of polyamines, which regulate growth and development in all living organisms (see section “AdoMet-Derived Polyamines and Ethylene Are Regulatory Metabolites in Stress Responses”). In plants, AdoMet is a precursor of the phytohormone ethylene and the iron chelator nicotianamine (NA) (Figure 3).

AdoMet is synthesized from Met and ATP by AdoMet synthetase (also known as Met adenosyltransferase and thus abbreviated as MAT). In mammals, MAT isoenzymes were classically considered to be cytosolic proteins (Pajares et al., 2013). However, they were also found in the nucleus, suggesting that they may function to provide AdoMet for the methylation of DNA and histones (Pajares et al., 2013). Similarly, AdoMet was previously considered to be synthesized only in the cytosol of plants (Ravanel et al., 2004). However, recent studies have shown that MAT isoenzymes are localized both in the nucleus and cytosol in *Arabidopsis* (Mao et al., 2015; Chen et al., 2016). Methylation reactions using AdoMet also occur in chloroplasts. AdoMet is imported from the cytosol to chloroplasts via SAM transporter 1 (SAMT1)/SAM career 1 (SAMC1), which is localized to the chloroplast envelope membranes and functions as an AdoMet–AdoHcy antiporter (Bouvier et al., 2006; Palmieri et al., 2006). As mentioned above, AdoHcy, a product of methylation reactions, needs to be transported to the cytosol and degraded by SAHH to form Hcy.

Hcy derived from AdoHcy degradation is regenerated into Met by MS in the cytosol of yeast, mammals, and plants (Figure 3). Consequently, the conversion of Met–AdoMet–AdoHcy–Hcy–Met forms the SAM cycle in cytosol. In addition, plants have another Met recycling system from Hcy called the *S*-methylmethionine (SMM) cycle, which involves Met *S*-methyltransferase (Met*S*MT) and Hcy *S*-methyltransferase (Hcy*S*MT) in the cytosol. Met*S*MT and Hcy*S*MT catalyze the conversion of AdoMet to AdoHcy and Hcy to Met, respectively, coupled with the conversion between Met and SMM (Sauter et al., 2013). SMM is a storage form of S and methyl groups in the leaves and a transport form of reduced S in the phloem (Bourgis et al., 1999). The SMM cycle plays a role in the maintenance of the AdoMet/AdoHcy ratio (Kocsis et al., 2003), which is thought to be a metabolic indicator of the methylation potential (Hoffman et al., 1979; Moffatt and Weretilnyk, 2001).

In plants, AdoMet is utilized for the biosynthesis of polyamines, NA, and ethylene. In these biosynthetic reactions, 5′-methylthioadenosine (MTA) is generated as a common by-product. MTA is a product inhibitor of spermidine synthase (SPDS), spermine synthase (SPMS), thermospermine synthase (TSPMS), NA synthase, and ACC synthase (ACS) involved in ethylene biosynthesis, and its accumulation is toxic. Then, MTA is detoxified and recycled via the Met salvage cycle (also known as the Yang cycle or MTA cycle) in the cytosol to generate Met (Sauter et al., 2013) (Figure 3). This cycle is common in plants, mammals, and bacteria, but involved enzymes seem to have been recruited from different metabolic pathways, depending on organisms during evolution (Sekowska et al., 2019). Unexpectedly, transcriptome analyses suggested that the MTA cycle genes in plant are specifically expressed in phloem. This hypothesis on phloem-specific expression was confirmed experimentally and further resulted in identification of 5-methylthioribose-1-phosphate isomerase 1 (MTI1) and dehydratase-enolase-phosphatase complex 1 (DEP1), which had been missing enzymes in plant (Pommerrenig et al., 2011). DEP1 is responsible for the conversion of 5-methylthioribulose-1-phosphate (MTRu-P) to 1,2-dihydro-3-keto-5-methylthiopentene (DHKMP) in plant, whereas in *Bacillus*, the conversion is catalyzed by three enzymes. Interestingly, one of three enzymes, 2,3-diketo-5-methylthiopentyl-1-phosphate enolase in a non-photosynthetic bacterium *Bacillus*, is an analog of ribulose bisphosphate carboxylase/oxygenase (RuBisCO) (Ashida et al., 2003).

### One-Carbon Metabolism Involves Tetrahydrofolate and AdoMet Metabolism

**FIGURE 4:**
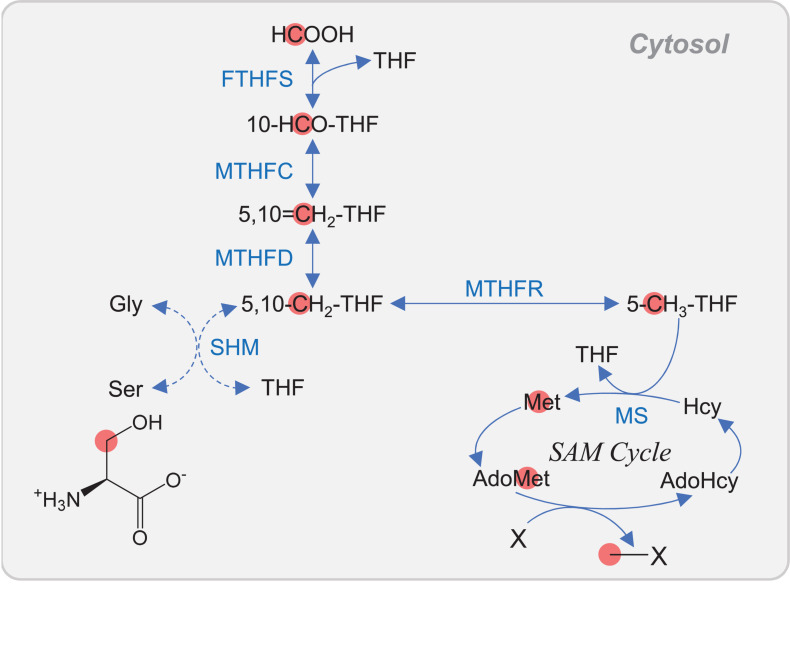
C_1_ metabolism in cytosol of plant. Carbon atoms of C_1_ unit are shown as red circles. Abbreviations are listed in Table 1. In *Arabidopsis*, the cytosolic bifunctional MTHFD/MTHFC is encoded by *MTHFD1*.

One-carbon (C_1_)-substituted folates are required for the biosynthesis of various important molecules. The methyl moiety of AdoMet is also derived from C_1_-substituted folates, and thus, C_1_ metabolism is essential in all living organisms. C_1_ derivatives of tetrahydrofolate (THF) are interconverted between different oxidation states, ranging from the most oxidized 10-formyl- (10-HCO-) THF through to 5,10-methenyl- (5,10=CH_2_-) THF and 5,10-methylene- (5,10-CH_2_-) THF to the most reduced 5-methyl- (5-CH_3_-) THF (Hanson et al., 2000) (Figure 4). The 5,10-CH_2_-THF is generated from the conversion of 5,10=CH_2_-THF catalyzed by 5,10-CH_2_-THF dehydrogenase (MTHFD). The 5,10-CH_2_-THF is also synthesized via the reversible reaction of Ser and THF to form Gly and 5,10-CH_2_-THF, which is catalyzed by Ser hydroxymethyltransferase (SHM). The conversion of 5,10=CH_2_-THF to 10-HCO-THF is catalyzed by 5,10=CH-THF cyclohydrolase (MTHFC). The 10-HCO-THF is used for the synthesis of purines and formylmethionyl-tRNA, which is used for translation initiation in plastids and mitochondria (Figure 1). The 5,10-CH_2_-THF is used for the synthesis of thymidylate and pantothenate. In eukaryotes, C_1_ folate metabolism is found in multiple organelles, including the mitochondria and cytosol of mammals, whereas in plants, it is also found in plastids (Hanson et al., 2000; Christensen and MacKenzie, 2006). However, MTHFR, which converts 5,10-CH_2_-THF to 5-methyl-(5-CH_3_-)THF, is found only in the cytosol (Hanson et al., 2000; Christensen and MacKenzie, 2006) (Figure 4). In the cytosol, Met is regenerated from Hcy via MS using 5-CH_3_-THF as a methyl donor in yeast, mammals, and plants (Christensen and MacKenzie, 2006). In *Arabidopsis*, a point mutation in the cytosolic bifunctional MTHFD/MTHFC (MTHFD1) resulted in the increase of THF + 5,10-CH_2_-THF (these two compounds were not distinguished by the analysis) and the decrease of 5,10=CH_2_-THF + 10-HCO-THF (these two compounds were also not distinguished) (Groth et al., 2016). Moreover, the mutation caused the accumulation of AdoHcy and Hcy, DNA hypomethylation, and loss of histone H3 lysine 9 methylation, indicating the interconnection of THF metabolism, AdoMet recycling, and DNA methylation. One of the major differences among the organisms is that C_1_ metabolism in mitochondria is related to photorespiration in plants (Figure 1). In the mitochondria of both photosynthetic and non-photosynthetic plant tissues, the major C_1_ donor is Gly via mitochondrion-specific Gly decarboxylase complex (GDC). To satisfy the demands of photorespiration, the flux through GDC and SHM reactions is in the direction to make Ser from Gly. In contrast, in yeast, Ser is the major C_1_ donor in the mitochondria, and the SHM reaction is reversible (Christensen and MacKenzie, 2006).

SHM is a conserved enzyme in living organisms from bacteria to higher plants and mammals and plays an important role in C_1_ metabolism (McClung et al., 2000; Hanson and Roje, 2001; Bauwe and Kolukisaoglu, 2003). In plant, the SHM isoenzymes are localized in the cytosol, mitochondria, plastids, and nucleus (Ruszkowski et al., 2018). The *SHM* gene family consists of seven genes in *Arabidopsis*. The mitochondrial SHM1 and SHM2 are involved in the photorespiratory Gly-into-Ser conversion (Voll et al., 2006; Engel et al., 2007), and the plastidic SHM3 catalyzes a reversible hydroxymethyl group transfer from Ser to THF, yielding Gly and 5,10-CH_2_-THF (Zhang et al., 2010). SHM7 in the nucleus lacks conventional SHM activity and is required for AdoMet production (see section “OAS Cluster Genes: OAS Functions as a Signal in Plants”).

Fluxes through the C_1_ pathways are particularly high in plants, because they are rich in methylated compounds such as lignin, alkaloids, and betaines (Hanson et al., 2000).

### Ser Metabolism Interconnects Metabolic Network

The proteinogenic amino acid Ser has a function as a precursor for various essential biomolecules such as nucleic acid bases, phospholipids, sphingolipids, and other amino acids in all organisms. In mammal, Ser metabolism plays crucial roles in, for example, brain development and function, as well as endothelial cell proliferation (Hirabayashi and Furuya, 2008; Vandekeere et al., 2018; Le Douce et al., 2020). In plants, Ser plays important roles not only in development (see below) but also in the response to environmental stresses, such as high salinity, flooding, and low temperature (Ho and Saito, 2001).

**FIGURE 5:**
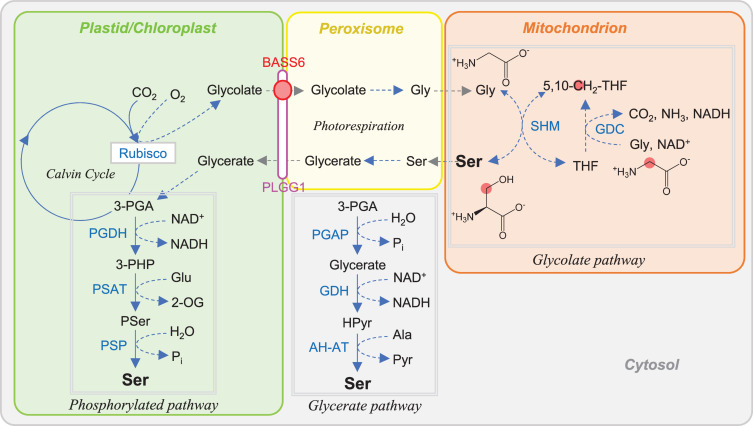
Ser biosynthesis in plant. Carbon atoms of C_1_ unit are shown as red circles. Abbreviations are listed in Table 1.

In contrast to the Asp-family amino acids that human must acquire from their diet, Ser can be synthesized in all living organisms including humans. Plants have three Ser biosynthetic pathways in different organelles: the glycolate pathway in mitochondria, the phosphorylated pathway in chloroplasts, and the glycerate (non-phosphorylated) pathway in cytosol (Figure 5).

The glycolate pathway forms a part of photorespiration processes (Figures 1, 5) and is a major source of Ser in photosynthetic organs. In photorespiration, a toxic metabolite phosphoglycolate is formed via the oxygenase activity of RuBisCO in chloroplast. This metabolite is recycled to 3-phosphoglycerate (PGA) in the sequential reactions occurring across chromoplast, peroxisome, and mitochondrion and turned back to the Calvin–Benson cycle. In the glycolate pathway, two Gly molecules are converted to one Ser molecule catalyzed by GDC and SHM in the mitochondria. Two transporters are involved in photorespiration processes: the plastidal glycolate glycerate translocator 1 (PLGG1) responsible for the simultaneous export of glycolate from and import of glycerate into the chloroplast (Pick et al., 2013) and the bile acid sodium symporter (BASS6) for export of glycolate from chloroplast (South et al., 2017).

The phosphorylated pathway of Ser biosynthesis, recently called PPSB, is common in plants, bacteria, and animals. In this pathway, PGA is converted to Ser via three-step reactions catalyzed by 3-PGA dehydrogenase (PGDH), 3-phosphoserine aminotransferase (PSAT), and 3-phosphoserine phosphatase (PSP) (Figure 5). In plants, these enzymes are localized in the plastid (Ho et al., 1998, 1999; Benstein et al., 2013; Cascales-Miñana et al., 2013; Toujani et al., 2013a; Wulfert and Krueger, 2018). In *Arabidopsis*, PGDH, PSAT, and PSP are encoded by three genes, two genes, and one gene, respectively. Most of the genes are dominantly expressed in heterotrophic tissues, except AtPGDH3 and AtPSP1 (Benstein et al., 2013), suggesting that PPSB supplies Ser to the heterotrophic tissues. However, PPSB also plays a role in the photosynthetic organs when photorespiration is inhibited at night or under high CO_2_ conditions (Ros et al., 2014). In *E. coli* and *Mycobacterium tuberculosis*, PGDH is feedback-inhibited by Ser (Pizer, 1963: Dey et al., 2005), whereas PGDH of human and rat is not (Achouri et al., 1997; Dey et al., 2008). In *Arabidopsis*, AtPGDH1 and AtPGDH3, but not AtPGDH2, are inhibited by Ser (Benstein et al., 2013; Okamura and Hirai, 2017). In a basal land plant *Marchantia polymorpha*, a single gene-encoded MpPGDH is inhibited by Ser (Akashi et al., 2018). In addition, AtPGDH1, AtPGDH3, and MpPGDH are activated by alanine, valine, Met, homoserine, and Hcy, suggesting interaction between PPSB and other amino acid metabolism (Okamura and Hirai, 2017; Akashi et al., 2018). AtPGDH1 and AtPSP1 are essential in pollen and embryo development (Benstein et al., 2013; Cascales-Miñana et al., 2013; Toujani et al., 2013b).

In the glycerate (non-phosphorylated) pathway, PGA is first dephosphorylated by PGA phosphatase and then subjected to the reactions catalyzed by glycerate dehydrogenase (GDH) and glycine:hydroxypyruvate (Ser:glyoxylate) and alanine:hydroxypyruvate (Ser:pyruvate) aminotransferases finally to form Ser (Igamberdiev and Kleczkowski, 2018). This pathway exists in the cytosol and is a major Ser source in the photosynthetic tissues of C_4_ plants, in darkness in C_3_ plants, and in non-photosynthetic tissues (Igamberdiev and Kleczkowski, 2018).

As mentioned in Section “OAS Is a Key Metabolite in Cys Synthesis,” Ser is converted to OAS by SERAT and reacts with sulfide to form Cys. On the other hand, Ser reacts with indole and directly forms tryptophan (Trp) being catalyzed by Trp synthase. Thus, Ser provides a carbon skeleton to both Cys and Trp. Ser is also involved in C_1_ metabolism via SHM (see section “One-Carbon Metabolism Involves Tetrahydrofolate and AdoMet Metabolism”). In this way, Ser metabolism interconnects S, Trp, and C_1_ metabolism.

## Regulatory Function of OAS, Adomet, Hcy, and Ser

The aforementioned pathways are mutually regulated by fine mechanisms that utilize key metabolites as signaling molecules. In the following chapters, we introduce the regulatory mechanisms involving four amino acids, OAS, AdoMet, Hcy, and Ser.

### OAS

Briefly, OAS has a signal function to regulate S-responsive gene expression. OAS also regulates Cys biosynthesis at enzymatic level.

#### OAS and *N*-Acetylserine Regulate Transcription in Bacteria and Plants

OAS is considered a signal molecule in plants because it is highly accumulated in S-deficient conditions, and OAS and/or its isomer *N*-acetylserine (NAS) acts as a positive regulator for the biosynthesis of Cys and S utilization in bacteria (Ostrowski and Kredich, 1991; Lynch et al., 1994). In bacteria, the Cys regulon, consisting of more than 20 genes, is coordinately controlled by a LysR-type regulator CysB (Kredich, 1996; Van Der Ploeg et al., 2001; Parry and Clark, 2002). OAS and/or NAS bind to CysB, activating its binding to the promoter of genes in the Cys regulon and preventing autorepression (Kredich, 1996). Although several results from previous studies have suggested that OAS is spontaneously converted to NAS in a pH-dependent manner (Flavin and Slaughter, 1967) and that NAS is the true signal for the Cys regulon (Ostrowski and Kredich, 1989; Hryniewicz and Kredich, 1994; Lynch et al., 1994), recent research of ligand recognition by CysB in *Salmonella typhimurium* has proposed that CysB has two distinct ligand-binding sites: site-1 for sulfate and NAS and site-2 for NAS and OAS. The OAS remodels site-1 by binding to site-2 to enhance the NAS-mediated activation through allosteric coupling between sites (Mittal et al., 2017). Furthermore, *N*,*O*-diacetylserine produced from OAS in *Salmonella enterica* may be the true signal for the Cys regulon (Vandrisse and Escalante-Semerena, 2018). More studies are required to fully confirm the activation mechanisms of CysB.

In plants, some studies have demonstrated that OAS and not NAS induced the expression of enzymes for the sulfate assimilation pathway (Neuenschwander et al., 1991; Hawkesford et al., 1995), which is the reason why OAS is considered a signal molecule in plants. However, the mechanisms for the transcriptional activation of S metabolism by OAS remain unclear in plants. Transcription regulators such as CysB or a receptor, which could be directory bound by OAS, have not yet been discovered.

#### OAS Cluster Genes: OAS Functions as a Signal in Plants

The role of OAS as a signal molecule in plants has been supported by the transcriptome analyses of sulfate starvation experiments (Hirai et al., 2003; Maruyama-Nakashita et al., 2003; Nikiforova et al., 2003), where OAS was accumulated in plants, and in OAS addition experiments (Koprivova et al., 2000; Kopriva et al., 2002; Hesse et al., 2003; Hopkins et al., 2005). The OAS concentration in *Arabidopsis* grown on full nutrient condition was approximately 1–10 nmol/g fresh weight in leaves and roots, whereas the concentration was increased by 10–100 times in S-starved plants (Maruyama-Nakashita et al., 2006; Hubberten et al., 2012a; Forieri et al., 2017). Hubberten et al. (2012b) succeeded in identifying OAS cluster genes (Table 2), which are specifically coexpressed with OAS accumulation, using metabolite–gene correlation analyses with time-series transcriptome and metabolome data in non-S-related experiments of *Arabidopsis*; the studies of natural diurnal oscillations (Espinoza et al., 2010) and responses to light and temperature (Caldana et al., 2011), where the OAS concentration was changed in the range from two to four times the baseline. Furthermore, they evaluated the OAS-correlated genes using inducible overexpression lines of SERAT (Hubberten et al., 2012b). The six OAS cluster genes were highly coexpressed in various experimental conditions in *Arabidopsis* (Obayashi et al., 2007, 2009; Mutwil et al., 2010) and in other plant species such as *Oryza sativa* and *Populus trichocarpa* (Mutwil et al., 2008; Mutwil et al., 2011). The promoter regions of the OAS cluster genes contained a conserved *cis*-element, UPE box (UP9 binding element; UPregulated by S deficit 9, see below), or UPE-like motif (Wawrzynska et al., 2010). Transcription factors in the ethylene-insensitive 3-like (EIL) family including S limitation 1 (SLIM1), which is a central transcriptional regulator of S responses in *Arabidopsis* (Maruyama-Nakashita et al., 2006), were reported to bind the UPE box (Wawrzynska et al., 2010).

**TABLE 2:** OAS cluster genes in *Arabidopsis*.

Gene name	AGI code	Annotation	Function	References
*APR3*	At4g21990	Adenosine-5′-phosphosulfate reductase 3	Sulfur assimilation	Vauclare et al., 2002
*ChaC/GGCT2;1*	At5g26220	ChaC-like protein/γ-glutamyl cyclotransferase	GSH degradation	Paulose et al., 2013; Joshi et al., 2019
*LSU1*	At3g49580	Low sulfur-induced 1		Garcia-Molina et al., 2017
*SDI1*	At5g48850	Sulfur deficiency-induced 1	glucosinolate biosynthesis repressor	Aarabi et al., 2016
*SDI2*	At1g04770	Sulfur deficiency-induced 2	glucosinolate biosynthesis repressor	Aarabi et al., 2016
*SHM7/MSA1*	At1g36370	Serine hydroxymethyltransferase 7/more sulfur accumulation1	*S*-adenosylmethionine production	Huang et al., 2016

Recent studies have shown that the OAS cluster genes are involved in S metabolism, although the function of APR3 has already been defined as a key-limiting enzyme in the assimilatory reduction of sulfate (Vauclare et al., 2002). The ChaC protein is considered to be a cation transporter because *ChaC* was identified as a gene of the Cha operon (Ca^2+^/H^+^ antiporter) in *E. coli* (Ivey et al., 1993; Osborne et al., 2004). However, Kumar et al. (2012) found that mammalian ChaC has γ-glutamyl cyclotransferase (GGCT) activity degrading glutathione (GSH) to yield 5-oxoproline and cysteinyl-glycine for the recycling of GSH (Kumar et al., 2012). In the *Arabidopsis* genome, there are three ChaC genes, which all have GGCT activity (Kumar et al., 2015). Studies with knockout mutants or overexpression lines of the *GGCT2;1*, an OAS cluster gene, revealed that the GSH recycling by GGCT2;1 is important for heavy metal toxicity such as arsenite and cadmium (Paulose et al., 2013) and root system architecture during S starvation (Joshi et al., 2019).

The *low sulfur-induced* (*LSU*) gene was originally identified in *Nicotiana tabacum* as an S-responsive gene, named *UP9* (Wawrzynska et al., 2005). *UP9C*, which is one of six *UP9* genes, is highly upregulated under S starvation (Lewandowska et al., 2005) and has been suggested to be involved in the synthesis and signaling of ethylene (Moniuszko et al., 2013) and autophagy (Zientara-Rytter et al., 2011) through protein–protein interactions. In the *Arabidopsis* genome, there are four *LSU* genes, of which three (*LSU1*, *2LSU*, and *LSU3*) are induced by S deficiency (Nikiforova et al., 2003, 2005a; Sirko et al., 2015), but only *LSU1* was identified as an OAS cluster gene. In the large-scale interactome studies of proteins, LSU proteins have been identified as network hubs coordinating abiotic stress responses (Garcia-Molina et al., 2017; Vandereyken et al., 2018). Garcia-Molina et al. (2017) experimentally confirmed that LSU1 plays an important role in disease resistance to several abiotic stresses including nutrient deficiency, high salinity, or heavy metals via interactions with the chloroplastic Fe superoxide dismutase FSD2 (Garcia-Molina et al., 2017).

The *sulfur deficiency-induced* (*SDI*) gene was originally identified in wheat (*Triticum aestivum* var. Hereward) grown in S-deficient fields as an indicator of S status by cDNA-amplified fragment length polymorphism analysis (Howarth et al., 2005). In the *Arabidopsis* genome, there are five *SDI* genes categorized in the *Male-sterile 5* (*MS5*) gene family, of which two *SDI* genes (*SDI1* and *SDI2*) belong to the OAS cluster genes. Studies with knockout mutants or overexpression lines of the *SDI* genes revealed that SDI1 and SDI2 proteins acted as major repressors controlling glucosinolate biosynthesis in *Arabidopsis* (Aarabi et al., 2016). Further experiments using protein–protein interaction assays demonstrated that SDI1 physically interacts with MYB28, which is a positive regulator of aliphatic glucosinolates (Gigolashvili et al., 2007; Hirai et al., 2007; Sønderby et al., 2007; Malitsky et al., 2008), to repress the glucosinolate biosynthesis and use the sulfate preferentially for primary metabolites under S deficiency conditions (Aarabi et al., 2016, 2020).

The *SHM7* gene was identified as the causal gene of the ethyl methanesulfonate-mutagenized *more sulfur accumulation1-1* (*msa1-1*) mutant (Huang et al., 2016). Characterization of SHM7 and the *msa1-1* mutant revealed that SHM7 lacks conventional SHM activity, is localized in the nucleus, and is required for AdoMet production and maintaining S homeostasis epigenetically via DNA methylation of, for example, the sulfate transporter genes *SULTR1;1* and *SULTR1;2* (Huang et al., 2016).

#### OAS Regulates the Cys Synthase Complex

SERAT (a dimer of homotrimers) and OAS-TL (a dimer) in the final steps of Cys biosynthesis form a hetero-oligomeric Cys synthase complex (CSC) (Kredich et al., 1969; Wang and Leyh, 2012). The CSC plays an important role in plant and bacterial S metabolism to strictly regulate the Cys concentrations in cells. The function for this CSC formation is not metabolic channeling. In the CSC, SERATs are activated or stabilized, whereas OAS-TLs are inactivated. OAS-TLs are fully active in the free form (Kredich et al., 1969; Droux et al., 1998; Mino et al., 2001). The balance of the complex and free forms of SERAT and OAS-TL is regulated by precursors of Cys synthesis, OAS and sulfide (Kredich et al., 1969; Droux et al., 1998; Mino et al., 2001). OAS dissociates the CSC, whereas sulfide is associated with CSC formation; hence, the association stimulates OAS formation, whereas the dissociation stimulates both OAS consumption and Cys formation. In addition to the regulation of CSC, SERAT activity is feedback inhibited by Cys (Kredich et al., 1969; Hindson, 2003; Olsen et al., 2004). The quaternary structures of the CSC from the bacteria and plants are highly similar, but the affinity of the CSC formation and the regulation of Cys sensitivity of SERAT within the CSC are different (Wirtz et al., 2010). For example, the affinity of CSC in *Arabidopsis* is 10 times weaker than that of bacterial CSCs in *S. typhimurium*, *Haemophilus influenzae*, and *E. coli*. The CSC formation decreases the sensitivity of SERAT to feedback inhibition by Cys in plants, whereas that of bacterial SERAT is not affected by the CSC formation. SERATs in bacteria such as *E. coli* (Kredich, 1996; Hindson, 2003; Olsen et al., 2004) and *S. typhimurium* (Kredich, 1996) are sensitive to feedback inhibition by Cys. The importance of CSC formation was well studied in *Arabidopsis* and soybean. Cytosolic and mitochondrial SERATs in *Arabidopsis* and cytosolic SERAT in soybean, which are feedback-sensitive by Cys, are less sensitive to the inhibition in CSC *in vitro* assay (Wirtz et al., 2010). Furthermore, the importance of CSC formation *in vivo* to regulate SERAT activity, and consequently, Cys production was proven by analyzing *Arabidopsis* T-DNA mutants of mitochondrial SERAT and OAS-TL (Wirtz et al., 2012). In plants, generally there are multiple SERAT isoforms localized in several subcellular compartments. The presence of Cys feedback regulation differs both between plant species and between subcellular compartments. For example, cytosolic SERAT isoforms in *C. vulgaris* (Saito et al., 1995) and *Allium tuberosum* (Urano et al., 2000) and plastidial isoforms in *S. oleracea* (Noji et al., 2001) and *P. sativum* (Droux, 2003) are sensitive to the feedback inhibition.

#### Transporters of OAS Are Necessary for Signaling Function

In bacteria, to act as signal molecules binding to a regulator CysB in the nucleus, OAS and/or NAS, which are biosynthesized in the cytosol, need to be transported into the nucleus. Ions and small metabolites are expected to diffuse through water-filled channels in the nuclear pore complex (Beck and Hurt, 2017). In contrast to the bacteria, OAS, which is presumed to be a true signal in plants, is synthesized in multiple compartments, including the cytosol, plastids, and mitochondria (Saito, 2004; Takahashi et al., 2011). Several previous investigations using *Arabidopsis* knockout mutants of SERATs and OAS-TLs have implied that OAS and Cys are transported between cellular compartments (Heeg et al., 2008; Lopez-Martin et al., 2008; Watanabe et al., 2008a, b; Krueger et al., 2009). However, the transport system is currently unclear in plants, although several transporters of OAS and Cys such as YdeD, YfiK, Bcr, and TolC, which export both compounds from the cytosol to the outside of the cells, have been identified in bacteria (Dassler et al., 2000; Franke et al., 2003; Koronakis et al., 2004; Yamada et al., 2006). The subcellular distribution of OAS is a key issue. It is not yet clear in which compartment OAS acts as a signal molecule, how OAS induces the OAS cluster genes, or whether the distribution of OAS between the compartments is limited or not. The identification of the transport system will be helpful to elucidate the mechanisms of gene induction by OAS and to understand why OAS and Cys synthesis takes place in multiple compartments in plants.

#### OAS-Related Metabolites Also Function as Signals in S Assimilation Pathway

In addition to OAS, other OAS-related metabolites such as sulfide, Cys, and GSH, which are interconnected in S assimilation pathway, are also considered to be possible signal molecules (Kopriva, 2006; Rouached et al., 2008). Sulfide, Cys, and GSH, which are reduced forms of S, significantly decrease sulfate uptake and sulfate assimilation via changing the activity of enzymes and the transcript levels of genes (Brunold and Schmidt, 1976; Lappartient et al., 1999; Westerman et al., 2001; Vauclare et al., 2002). In S starvation conditions and feeding of each compound, the concentrations of these possible signal molecules were changed all together in plants, leading to disagreement regarding which metabolites were the true signals. Recent studies progressively revealed the specific function of each metabolite as a signal molecule. Sulfide has been proven to be involved in protein persulfidation causing changes in the protein functions (Aroca et al., 2018). Cys has been reported to stimulate the synthesis of hormone abscisic acid (ABA) via inducing the transcript level of 9-*cis*-epoxycarotenoid dioxygenase 3 (NCED3), which is the key rate-limiting enzyme for ABA production (Batool et al., 2018). Dong et al. (2017) showed that *Arabidopsis* senses the availability of OAS as the carbon/nitrogen precursor for Cys synthesis with the general control non-derepressible 2 (GCN2) kinase and the availability of sulfide as the S precursor for Cys synthesis with the target of rapamycin by downregulation of glucose metabolism. The identification of distinct signal function of each metabolite could help to understand the whole framework of S starvation responses of plants.

### AdoMet

The most important feature of regulatory function of AdoMet is that it could control epigenomic code via DNA/histone methylation in development and stress responses.

#### AdoMet Is a Critical Biomolecule Controlling Enzymatic Activity and Gene Expression at the Level of Transcription and Translation

AdoMet is recognized as an active form of Met and is one of the most important S-containing metabolites in plants. AdoMet is a key regulator of the Asp pathway, a major methyl donor for various methylation reactions, and a precursor of the polyamine, ethylene, and NA biosynthesis.

AdoMet is directly synthesized from Met and thus a critical biomolecule for regulation of the biosynthesis of Met and other amino acids in the Asp family. AdoMet acts as an allosteric effector for inhibition of Asp kinase and activation of Thr synthase (Azevedo et al., 1997; Mas-Droux et al., 2006) (Figure 1). While most of the key steps in the Asp pathway are regulated by allosteric enzymes, CGS, which catalyzes the first committed step of Met biosynthesis, is not one of them. CGS is functional in chloroplasts; however, AdoMet biosynthesis occurs in the cytosol. CGS is a chloroplast protein encoded by the nuclear *CGS1* gene in *Arabidopsis*. Its expression is regulated via negative feedback by temporal translation elongation arrest, followed by mRNA degradation in response to AdoMet (Chiba et al., 1999, 2003; Onouchi et al., 2005) (at the bottom right of Figure 1). *Arabidopsis mto1* mutants, which overaccumulate soluble Met, are defects in this posttranscriptional regulation. The *mto1* mutations are found as single amino acid changes in the short stretch of the CGS1 nascent peptide, called the MTO1 region. The MTO1 region acting as a *cis* element is necessary and sufficient for the feedback regulation of CGS1. The temporal translation arrest occurs at the Ser-94 codon immediately downstream of the MTO1 region. During the translation arrest, the CGS1 nascent peptide including the MTO1 region forms a compact conformation inside the ribosomal exit tunnel. Furthermore, the compaction is associated with the conformation changes of the rRNA in the ribosomal exit tunnel, which might be involved in the translational arrest of the *CGS1* gene in response to the AdoMet (Onoue et al., 2011). To maintain the homeostasis of the AdoMet produced in the cytosol, the translational feedback regulation of CGS1 in the cytosol is thought to be an appropriate system by which to achieve a prompt response to AdoMet.

AdoMet is a universal methyl group donor for various methylated metabolites. After the methyl group is transferred by methyltransferases to the accepted substrate, AdoHcy is produced and then rapidly hydrolyzed to Hcy and adenosine by SAHH, because AdoHcy is a strong inhibitor of all AdoMet-dependent methyltransferases. The AdoMet/AdoHcy ratio is thought to be a metabolic indicator of the methylation potential (Hoffman et al., 1979; Moffatt and Weretilnyk, 2001). DNA and histone proteins can be target for methylation. Their methylation status acts as an epigenetic code, which then influence the regulation of gene expression. In *Arabidopsis*, the steady-state levels of AdoMet and AdoHcy are reported to be ∼15 and ∼0.5 pmol/mg fresh weight, respectively, in rosette leaves (Groth et al., 2016), and 3.34 and 0.14 pmol/mg, respectively, in leaves (Rocha et al., 2005). The AdoMet/AdoHcy ratio varies according to conditions. For example, under S deficiency, AdoMet significantly decreased in seedlings, whereas AdoHcy remained unchanged, resulting in the decrease of the AdoMet/AdoHcy ratio (Nikiforova et al., 2005b). In another study, AdoMet in root significantly decreased, and DNA methylation pathway genes were downregulated under S deficiency, whereas AdoHcy also decreased to a greater extent than AdoMet, thus resulting in the increase of the AdoMet/AdoHcy ratio (Forieri et al., 2017). The study suggested that AdoMet amount itself is also important for methylation status. The AdoMet/AdoHcy ratio was also changed under iron (Fe) deficiency: the values were approximately 60 (control), 130 (S deficiency), and 180 (Fe deficiency) (calculated using the data from Forieri et al., 2017).

AdoMet/AdoHcy ratio is also important in mammals. Histone methyltransferases (HMTs) are enzymes that catalyze the transfer of methyl groups to histone proteins. Alterations to AdoMet-related metabolism affect the histone methylation status, suggesting a possible link between the AdoMet/AdoHcy ratio and HMT activity. These metabolic changes also lead to widespread secondary effects on stress responses and developmental pathways, which make it difficult to determine whether these changes on histone methylation are caused by the direct sensing of the AdoMet/AdoHcy ratio in the cells (Shyh-Chang et al., 2013; Ulanovskaya et al., 2013; Shiraki et al., 2014). However, recent studies have shown the direct effects of the AdoMet/AdoHcy ratio on histone methylation levels by demonstrating the kinetics of histone methylation turnover in response to AdoMet and AdoHcy availability (Mentch et al., 2015). As most of the HMTs are involved in the methylation of specific target genes, the metabolic status of AdoMet is critical for these gene-specific regulations.

In bacteria, AdoMet is an important metabolite, not only as a methyl group donor but also as an effector of riboswitches for regulating intracellular Met and AdoMet concentrations. Three evolutionarily distinct classes of AdoMet-binding riboswitches, the SAM-I, SAM-II, and SAM-III superfamilies, have been identified, of which the SAM-I riboswitch is widespread and the best studied in bacteria (Batey, 2011). The SAM-I riboswitches have been identified in many transcripts encoding enzymes involved in sulfate assimilation and Cys/Met biosynthesis, which include AdoMet biosynthesis itself (Grundy and Henkin, 1998). AdoMet is synthesized from Met and ATP by MAT and is converted to AdoHcy after the methyltransferase reaction. AdoHcy is hydrolyzed to Hcy and adenosine by SAHH, and then, Hcy is recycled back to Met by MS. SAM-I, SAM-II, and SAM-III riboswitches were identified in the MAT, and SAM-I and SAM-III were in MS (Batey, 2011). The negative-feedback regulation of these enzyme genes by the AdoMet-binding riboswitch could be required for the proper maintenance of the intracellular AdoMet level.

#### AdoMet Metabolism and DNA/Histone Methylation Are Involved in Development and Stress Responses

As described in Section “AdoMet Is a Critical Biomolecule Controlling Enzymatic Activity and Gene Expression at the Level of Transcription and Translation,” the AdoMet concentration and the AdoMet/AdHcy ratio in cells are related to the proper methylation potential of DNA and histone to maintain normal development. The studies using *Arabidopsis* mutants indicated that the AdoMet and folate metabolism affects the genome-wide DNA methylation level. The leaky mutants of SAMS3/MAT4 and SAHH1 (responsible for AdoMet synthesis and AdoHcy hydrolysis, respectively) and MTHFD1 (involving interconversion of folates) exhibited the genome-wide DNA demethylation and the reduced AdoMet/AdoHcy ratio, although the causal relationship between DNA demethylation and the reduced AdoMet/AdoHcy ratio remained to be further examined (Goto et al., 2002; Rocha et al., 2005; Groth et al., 2016; Meng et al., 2018). The knockout mutants of SAMS3/MAT4, SAHH1, and MTHFD1 genes were lethal, indicating that these genes are essential (Rocha et al., 2005; Groth et al., 2016). Adenosine kinase (ADK) catabolizes adenosine and thus enhances AdoHcy hydrolysis via SAHH (Figure 3; see section “Metabolism and a Putative Regulatory Role of Hcy”). In the ADK-deficient lines, AdoHcy increased by 12–43-fold, and the AdoMet/AdoHcy ratio was greatly reduced (AdoMet 17.39 μM and AdoHcy 0.34 μM in wild-type leaves) (Moffatt et al., 2002). The content of methyl-esterified pectin in seed mucilage was decreased with the reduced ADK activity. Considering morphological abnormality of the ADK-deficient lines, ADK plays an important function to AdoMet recycling and various methyltransferase reactions (Moffatt et al., 2002).

DNA methylation is involved in the various biotic and abiotic responses in plants. For instance, genome-wide DNA hypermethylation was observed in *Arabidopsis* roots in response to cyst nematode infection (Hewezi et al., 2017). Infection of bacterial pathogen also induced widespread differential DNA methylation in *Arabidopsis* leaves, which is negatively correlated with the expression of nearby transcripts (Dowen et al., 2012). In the case of abiotic stresses, early studies demonstrated abiotic stress-induced changes in DNA methylation patterns on genome-wide or specific regions, which are associated with transcriptional regulation of stress-responsive genes in some cases (Xu et al., 2015; Yong-Villalobos et al., 2015; Zhang et al., 2016). Afterward, research interest shifted to the epigenetic memory for abiotic stress responses in plants (Kinoshita and Seki, 2014; Chang et al., 2020). These studies suggest the interplays between the AdoMet and folate metabolism and epigenetic regulation that are important for plant stress adaptation. Recently, Gonzalez and Vera (2019) demonstrated that folate metabolism is key in the epigenetic regulation for plant immune system by elegant approach combining chemical and reverse genetic strategies.

Nutritional stresses alter metabolism and gene expression. Among them, Fe deficiency greatly affects S metabolism because Fe forms Fe-S clusters involved in electron transfer chains in mitochondria and chloroplasts (Balk and Schaedler, 2014). NA is a chelator of metal cations including Fe ions and synthesized by trimerization of three molecules of AdoMet (Figure 3). NA plays an important role in the internal transport of Fe, which is critical for proper reproductive development of plants (Takahashi et al., 2003). Fe deficiency resulted in increase of reduced S-containing metabolites including AdoMet, which might further result in higher accumulation of NA (Forieri et al., 2017). Also, a significant increase of the AdoMet/AdoHcy ratio under Fe deficiency might contribute to the large perturbation of transcription (Forieri et al., 2017).

#### AdoMet-Derived Polyamines and Ethylene Are Regulatory Metabolites in Stress Responses

AdoMet is also involved in stress responses through AdoMet-derived metabolites, such as polyamines and ethylene.

Polyamines are polycationic compounds with strong binding abilities for negatively charged biomacromolecules, such as acidic proteins, membrane phospholipids, and nucleic acids. Predominant forms of the polyamines in plants are diamine putrescine, triamine spermidine, and tetraamine spermine (Figure 3). Biosynthetic pathways for polyamines have been extensively studied in *Arabidopsis* (Hanfrey et al., 2001). The initial step of polyamine biosynthesis is decarboxylation of arginine (Arg) by Arg decarboxylase (ADC), and then two additional successive steps produce putrescine. Spermidine and spermine are synthesized by sequential additions of aminopropyl groups to putrescine and spermidine by SPDS and SPMS, respectively. These aminopropyl moieties are derived from dAdoMet produced by AdoMet decarboxylase (AdoMetDC) (Figure 3). Thermospermine, a structural isomer of spermine, is also produced from spermidine and dAdoMet by TSPMS. Interestingly, polyamine levels are increased in response to various abiotic stresses, such as salinity, drought, low and high temperatures, and nutrient deficiencies, suggesting a protective role for polyamines against environmental stresses (Groppa and Benavides, 2007; Alcázar et al., 2010). These responses are mostly due to the induction of gene expressions of polyamine biosynthesis genes under stress conditions (Alcázar et al., 2006, 2010; Cuevas et al., 2008). Especially, spermidine and spermine levels rely on the supply of dAdoMet. Elevated levels of spermine produced by the overexpression of *AdoMetDC* or *SPMS* genes in *Arabidopsis* enhanced the tolerance to drought and salt stresses (Gonzalez et al., 2011), whereas the *tspms*/*spms* double mutant showed opposite phenotypes due to reduced levels of spermine (Yamaguchi et al., 2006, 2007). Furthermore, transcriptome analyses using *AdoMetDC* or *SPMS* overexpressing plants demonstrated the inductions of several genes encoding key components in the stress signaling cascades, as well as a key enzyme of ABA biosynthesis, NCDE3, with spermine overaccumulation (Marco et al., 2011). The involvement of AdoMet-dependent polyamine biosynthesis in stress responses has been revealed in many plant species other than *Arabidopsis* via genetic engineering. There are many examples showing that transgenic plants, such as rice, tobacco, and tomato, with the *AdoMetDC* gene from different species have acquired greater tolerances to various environmental stresses (Roy and Wu, 2002; Waie and Rajam, 2003; Cheng et al., 2009).

Ethylene is an important phytohormone involved in multiple physiological and morphological responses, such as inhibition of cell growth, induction of fruit ripening and abscission, and adaptation to environmental stresses. Its biosynthesis pathway consists of a simple two-step reaction: AdoMet is converted to 1-aminocyclopropane-1-carboxylic acid (ACC) by ACS, and then ACC is converted to ethylene by ACC oxidase (Figure 3). Ethylene biosynthesis is accelerated in response to various biotic and abiotic stresses, such as pathogen infection, osmotic, salt, and drought stresses. Under salt stress, plants must control reactive oxygen species (ROS) level to survive. ROS act as a signal molecule to acquire the salt stress tolerance, whereas excess ROS accumulation damages cellular structure (Qi et al., 2018). Ethylene has a dual role to modulate salt stress response: one is promoting ROS production and signaling to activate Na^+^/K^+^ transport (Jiang et al., 2013), and the other is inducing the expression of ROS scavengers through ethylene signaling pathway (Peng et al., 2014). Supply of AdoMet is crucial for ethylene biosynthesis. Induction of MAT genes in response to abiotic stresses has been reported in several species, such as barley, sugar beet, cucumber, and tomato (Sánchez-Aguayo et al., 2004; Ma et al., 2017; He et al., 2019). In the case of barley, HvSAMS3 protein accumulation in leaves was enhanced under combined drought and salt stresses. The *hvsams3* knockdown mutants reduced tolerance to the combined stress, which was associated with decreased level of ethylene and polyamines. Moreover, exogenous application of ethylene to the mutants improved the tolerance, suggesting that ethylene level relying on HvSAMS3 is important for acquisition of the tolerance to combined stresses at least in part (Ahmed et al., 2020).

Overall, these studies indicate that AdoMet is an important precursor of polyamine and ethylene and leads to stress tolerance in plants.

### Hcy

Hcy metabolism seems to be influenced by environmental stresses, although there are few reports on its steady-state level owing to technical difficulty of measurement. Hcy regulates PGDH activity *in vitro* and thus may control Ser biosynthesis *in vivo*.

#### Metabolism and a Putative Regulatory Role of Hcy

As mentioned in Section “Ser Metabolism Interconnects Metabolic Network,” the enzymatic activity of AtPGDH1, AtPGDH3, and MpPGDH is activated by Hcy *in vitro* with the lowest EC_50_ values among the other activator amino acids (23 μM for AtPGDH1; 69 μM for AtPGDH3; ∼20 μM for MpPGDH) (Okamura and Hirai, 2017; Akashi et al., 2018). In *Arabidopsis*, the steady-state levels of Hcy are < 1 pmol/mg fresh weight in rosette leaves (Groth et al., 2016). In the hypocotyls of yellow lupine (*Lupinus luteus*) seedlings, the concentration of total Hcy (including disulfide-bound forms) was 4.3 μM (Jakubowski and Guranowski, 2003). Under S deficiency, the relative abundance of Hcy in *Arabidopsis* seedling was not significantly changed (Nikiforova et al., 2005b). On the other hand, perturbation of folate metabolism affected Hcy level: a point mutation in MTHFD1 resulted in the increase of Hcy to ∼7 pmol/mg fresh weight in *Arabidopsis* rosette leaves (Groth et al., 2016).

In mammals, Hcy is generated by hydrolysis of AdoHcy. Hcy is then converted to Cys via the transsulfuration pathway catalyzed by CysT β-synthase and CysT γ-lyase, which is the reverse reaction of the *de novo* Hcy biosynthesis (catalyzed by CGS and CBL) in plants. Otherwise, Hcy is remethylated to form Met via MS (using 5-CH_3_-THF as the methyl donor) or betaine–Hcy*S*MT (using betaine as the methyl donor). Hcy is considered toxic because its reduction power is stronger than that of Cys. Hcy is metabolized to Hcy-thiolactone by methionyl-tRNA synthetase (MetRS) in an error-editing reaction that prevents the translational incorporation of Hcy into proteins (Jakubowski, 2000; Jakubowski and Guranowski, 2003). Hcy-thiolactone synthesis is also found in bacteria and plants (see below). It is chemically reactive as its carbonyl group reacts with epsilon-amino group of Lys residues in proteins to form an amide bond (Hcy-*N*-protein), which leads to protein damage (Jakubowski, 2000; Jakubowski and Guranowski, 2003).

Overall, there is relatively little information available for Hcy in plants, and this is probably because it has been considered an intermediate of Met biosynthesis and a by-product of AdoMet metabolism. In contrast to its metabolism in animals, Hcy is the direct precursor of Met in plants and is synthesized in the chloroplast from Cys through the transsulfuration reactions catalyzed by CGS and CBL (see Asp pathway in Figure 1). Hcy is also generated from AdoHcy via SAHH reactions in the cytosolic AdoMet and SMM cycles (Figure 3). Because AdoHcy is generated as a by-product of various AdoMet-dependent methylation reactions and inhibits methyltransferase activities, it must be efficiently removed by SAHH. *Arabidopsis* possesses two SAHH isoforms, SAHH1 and SAHH2, and the null mutation of SAHH1 resulted in embryonic lethality (Rocha et al., 2005), indicating the importance of removal of AdoHcy. Biochemical analysis suggested that SAHH forms oligomeric protein complexes in phylogenetically divergent land plants and that light stress regulates protein complex formation and phosphorylation of SAHH, although the responses differed between *Arabidopsis* and *Physcomitrium patens* (Alegre et al., 2020).

SAHH also catalyzes the reverse reaction (i.e., AdoHcy synthesis), and the equilibrium is driven toward AdoHcy hydrolysis by the removal of the hydrolysis products, Hcy and adenosine (Guranowski and Pawełkiewicz, 1977; Pereira et al., 2007). Hcy is recycled in the SAM and SMM cycles (Figure 3), whereas adenosine is recycled predominantly by ADK; thus, SAHH and ADK are coordinately regulated developmentally and responding to environmental stresses (Pereira et al., 2007). For example, SAHH activity was increased by elicitation of the phytoalexin response in alfalfa cell culture (Edwards, 1996). The transcript levels of SAHH and ADK increased with increased NaCl concentration in spinach, probably to meet increased demand of AdoMet for the synthesis of Gly betaine, a compatible osmolyte (Weretilnyk et al., 2001). The SAHH transcripts increased in response to a UV stimulus in parsley (Logemann et al., 2000).

Jakubowski and Guranowski (2003) reported that Hcy-thiolactone is synthesized in plants. In the hypocotyls of yellow lupine (*L. luteus*) seedlings, Hcy-thiolactone was detected at a concentration of < 0.6 μM. Treatment with the antifolate drug aminopterin, which inhibits the methylation of Hcy to Met, enhanced the accumulation of Hcy (245 μM) and the synthesis of Hcy-thiolactone. The level of Hcy-*N*-protein was also increased after the aminopterin treatment. The Met supplement inhibited Hcy-thiolactone synthesis in yellow lupine seedlings, suggesting the involvement of MetRS. The *E. coli* cells expressing rice (*O. sativa*) MetRS were found to catalyze the conversion of Hcy to Hcy-thiolactone. The Hcy-thiolactone-hydrolyzing enzyme was purified from yellow lupine seed meal, suggesting Hcy-thiolactone metabolism in plant.

### Ser

Ser metabolism has important functions in development, although it remains to be clarified whether a regulatory molecule is Ser itself or Ser-related metabolites. Ser metabolism is also involved in stress responses such as salt tolerance and photosynthetic performance.

#### Ser Metabolism Affects S Metabolism

As mentioned in Section “Ser Metabolism Interconnects Metabolic Network,” Ser metabolism interconnects S, Trp, and C_1_ metabolism. In this section, we focus on the relationship between Ser biosynthesis and S metabolism, because Ser is the direct precursor of OAS production.

The OAS formation by SERAT is an interface between Ser as a nitrogen/carbon source and Cys metabolism as an S source. Therefore, the production of Ser affects that of OAS and then S metabolism. Although a variety of mutants in Ser biosynthesis have been reported and showed a complexity of metabolite changes independently of the Ser levels with growth phenotypes (Collakova et al., 2008; Benstein et al., 2013; Cascales-Miñana et al., 2013; Kuhn et al., 2013; Toujani et al., 2013a), a recent study by Anoman et al. (2019) revealed that deficient lines of the two genes in the phosphorylated pathway (*AtPGDH1* and *AtPSP1*) perturbed S homeostasis by regulating not only its biosynthesis, but also the transport and allocation of the thiols between photosynthetic and non-photosynthetic tissues. Specifically, the OAS level increased in both aerial parts and roots of *AtPSP1-* and *AtPGDH1*-deficient lines compared to controls (up to 2.7- and 4.2-fold the wild-type level, in the aerial parts and roots of *AtPSP1-*deficent line). Interestingly, in the presence of Ser in the growing medium, the OAS levels reverted to normal values (Anoman et al., 2019). On the other hand, in the *Arabidopsis* photorespiratory “*a bout de soufflé*” (*bou-2*) mutant, with reduced activity of the mitochondrial GDC multienzyme due to knockdown of mitochondrial BOU transporter protein, Ser was increased by fourfold (day) and 10-fold (night), whereas Cys was increased by fivefold (day) and threefold (night), compared to wild type (Samuilov et al., 2018).

The three Ser biosynthetic pathways are strictly regulated in terms of timing, tissue specificity, and transport of Ser between compartments, which results in some limitations to compensate for the loss of Ser in either compartment (Voll et al., 2006; Engel et al., 2011; Ros et al., 2014). In the case of *Arabidopsis*, the Ser concentration in the plants seems not to be affected by changes in the downstream products of S metabolism, such as OAS, Cys, GSH, or others (Watanabe et al., 2018). This might be caused by the bigger pool sizes of Ser compared to those of S metabolites or the tight regulation of Ser production by homeostatic mechanisms, including a feedback inhibition of, for example, AtPGDH1 activity by Ser and an activation by S-containing amino acids such as Cys, Hcy, and Met (Okamura and Hirai, 2017; Akashi et al., 2018). The EC_50_ values of AtPGDH1, AtPGDH3, and MpPGDH against Ser are 6.6, 1.3, and 1.5 mM, respectively. These values are likely within the biological range of Ser concentration in plastid, because Ser was present at 1.32 mM in chloroplasts from pea leaves (Mills and Joy, 1980).

The expression of *AtPGDH1* is positively regulated by MYB34 and MYB51, the transcription factors regulating the biosynthetic genes of Trp and Trp-derived indole glucosinolates (Benstein et al., 2013). In addition, Trp-derived metabolites such as indole acetic acid and indole glucosinolates were decreased in *AtPGDH1*-silenced plants, indicating that AtPGDH1 is involved in supplying the Ser used as the precursor of Trp biosynthesis (Benstein et al., 2013). The activation of AtPGDH1 activity by the S-containing amino acids may have a role to balance S and Trp metabolism.

#### Ser Metabolism Is Involved in Development and Environmental Adaptation

Ser metabolism is involved in adaptive responses to various environmental stresses. Plants subjected to low temperature and elevated salinity accumulate Ser (Ho and Saito, 2001). In certain halophytes, Ser is a precursor of ethanolamine, which is further converted to a compatible solute Gly betaine. A recent study showed that Ser was increased by threefold in aerial part and by eightfold in root of *Arabidopsis* under salt stress (Rosa-Tellez et al., 2020). In this case, the expression of *AtPGDH1* and *AtPGDH2* was induced, whereas that of *AtPGDH3* was repressed (Rosa-Tellez et al., 2020). This result indicated the isoform-specific functions of PGDH, although their roles in Ser accumulation remain to be clarified. Similarly, in *Beta vulgaris*, *BvPGDHa* was induced, whereas *BvPGDHb* was repressed under salt stress (Kito et al., 2017).

In *Arabidopsis*, each isoform of the phosphorylated pathway enzymes seems to have its specific function in development and stress responses. Among AtPGDHs, only the knockout of *AtPGDH1*, which was previously identified as the *embryo sac development arrest 9* (*EDA9*) gene, results in embryonic lethality (Benstein et al., 2013; Toujani et al., 2013a). *AtPGDH1* and *AtPGDH2* are regulated by MYB51, whereas *AtPGDH3* is not (Benstein et al., 2013). Similarly, *AtPSAT1*, but not *AtPSAT2*, is regulated by MYB51 (Benstein et al., 2013). Also, the expressions of *AtPGDH1*, *AtPGDH2*, and *AtPSAT1* are induced by infection of *Botrytis* (Benstein et al., 2013).

Because Ser biosynthesis involves NADH production (Figure 5), it is likely to affect NAD(P)^+^/NAD(P)H homeostasis and redox status of cell. In chloroplast, the reducing power is generated by photochemical reactions in the thylakoid membrane upon illumination. The redox cascade involving ferredoxin and thioredoxin ensures light-responsive coordination of chloroplast functions (Yoshida et al., 2020). AtPGDH1 is the target of redox regulation of chloroplast proteins, and this regulation is associated with the redox-active Cys pair uniquely found in land plant PGDH (Yoshida et al., 2020). Recently, Höhner et al. (2021) reported that AtPGDH3 plays a crucial role in NADH supply in plastid stroma and eventually in photosynthetic performance under natural fluctuating light environment.

The phosphorylated pathway plays a role in Ser supply in the photosynthetic organs when photorespiration is inhibited at night or under high CO_2_ conditions (Ros et al., 2014). Among three AtPGDHs, only *AtPGDH1* expression was induced under high CO_2_ conditions (Benstein et al., 2013).

In all above cases, the regulation of PGDH at the transcriptional or enzymatic levels is in an isoform-specific manner. Because the basal land plant *M. polymorpha* has a single PGDH, land plants may have evolved various types of PGDH after gene duplication during evolution to cope with various environmental stresses.

## Summary and Future Perspectives

In this review, we summarized the metabolism of S-containing amino acids and related compounds and the C_1_ metabolism in plants, focusing on regulatory function of AdoMet, OAS, Hcy, and Ser, in comparison with those in bacteria and animals. The following are some aspects that should be addressed in the future, although some of them have been topics of discussion for a long time.

- It remains unclear why some enzymes exist redundantly in several compartments (e.g., SERAT and OAS-TL in the plastids, mitochondria, and cytosol). On the contrary, some enzymes are localized exclusively to specific compartments. For example, SAHH, which hydrolyzes AdoHcy, exists only in the cytosol, although AdoHcy is generated as a by-product by methyltransferase reactions in the cytosol, plastids, and nuclei, and inhibits methyltransferase activities.

- The above issue might be related to our insufficient understanding of enzyme localizations. Alternative splicing or alternative promoter usage may regulate subcellular localization of enzymes. For example, glycerate kinase, which is an essential enzyme for photorespiration and targeted to the chloroplast, is localized to the cytosol under shaded condition by phytochrome-mediated alternative promoter selection. It is a part of cytosolic photorespiratory bypass that alleviates fluctuating light-induced photoinhibition (Ushijima et al., 2017). The regulation of protein subcellular localization, which might be realized by translational regulation, is the issue we should tackle.

- Although several metabolic pathways function across organelles, not many transporters involved in the intercompartmental transport of metabolites have been identified. Considering previous efforts to find transporters, novel strategies for their identification are necessary. Bioinformatics and deep learning approaches may enable us to predict candidate genes. Transport mechanism of metabolites could be clarified by uptake experiments using isolated organelle, as is the case with Lee et al. (2014), where careful inspection of Cys uptake kinetics indicated the existence of multiple specific Cys transporters in the mitochondrial membranes. Such an approach is useful to identify transporters.

- The concentration of metabolites in each compartment is key for the regulation of metabolism. However, their measurements are difficult, because metabolites are rapidly converted to other metabolites by enzymatic and non-enzymatic reactions during the isolation of organelles for metabolite analyses. Metabolomics technology for subcellular-level analysis is required. Non-aqueous fractionation is a powerful technique to study the subcellular compartmentation of metabolites in three main cellular compartments, cytosol, plastids, and vacuoles. Krueger et al. (2009) clarified the distribution of S containing metabolites including sulfate, sulfide, OAS, Cys, γ-glutamyl Cys, and GSH in *Arabidopsis* using this method. Immunogold labeling method is also useful to study the metabolite distribution. Koffler et al. (2011) clarified the distribution of GSH precursors including Cys, γ-glutamyl Cys, glutamate, Gly in cytosol, plastids, mitochondria, nuclei, peroxisomes, and vacuoles in *Arabidopsis*.

- Furthermore, it must be considered that the metabolites may be non-uniformly present in the cell due to the generation of so-called membraneless organelles by phase separation. This is related to the concept of metabolon, in which metabolites are directly transferred from an enzyme to others in the sequential enzymatic reactions. In addition to the research to investigate protein condensates, live imaging of metabolites may help understand this issue.

It has long been known that amino acids regulate the activities of their biosynthetic enzymes in a feedback manner. As shown in this review, amino acids also regulate metabolism at translational and transcriptional levels, reflecting the status of internal and external environments. A recent study demonstrated that perturbation in Arg level results in stunted gametophore morphology in *P. patens* (Kawade et al., 2020). Arg itself or a related metabolite might be the metabolite, which directly regulates development.

In conclusion, with rapid advances in experimental technology, new regulatory mechanisms of metabolism and development will be discovered in which metabolites involved in amino acid metabolism act as signal molecules.

## Author Contributions

MW, YC, and MYH wrote the manuscript. All authors contributed to the article and approved the submitted version.

## Conflict of Interest

The authors declare that the research was conducted in the absence of any commercial or financial relationships that could be construed as a potential conflict of interest.
